# Degree of Coupling in Microwave-Heating Polar-Molecule Reactions

**DOI:** 10.3390/molecules28031364

**Published:** 2023-01-31

**Authors:** Xingpeng Liu, Heping Huang, Linsen Yang, Kama Huang

**Affiliations:** 1 School of Network and Communication Engineering, Chengdu Technological University, Chengdu 611730, China; 2 College of Electronics and Information, Southwest Minzu University, Chengdu 610041, China; 3 College of Electronics and Information Engineering, Sichuan University, Chengdu 610064, China

**Keywords:** microwave heating, degree of coupling, the entropy-balance equation

## Abstract

Microwave-assisted chemical reactions have been widely used, but the overheating effect limits further applications. The aim of this paper is to investigate the coupling degree of the electromagnetic field and thermal field in microwave-heating chemical reactions whose polarization changes as the reactions proceed. First, the entropy-balance equation of microwave-heating polar-molecule reactions is obtained. Then, the coupling degree of the electromagnetic field and the thermal field in microwave-heating polar-molecule reactions is derived, according to the entropy-balance equation. Finally, the effects of reaction processes on the degree of coupling are discussed. When the time scale of the component-concentration variation is much greater than the wave period during the chemical processes, the degree of coupling is sufficiently small, and the electric and thermal fields are considered as weakly coupled. On the other hand, the degree of coupling may change during the reactions. If the absolute value of the coupling degree becomes larger, due to the change in component concentration, this will lead to a transition from weak coupling to strong coupling.

## 1. Introduction

Microwave heating has been used increasingly in the chemical industry. Compared with traditional heating, microwave heating has many advantages, such as acceleration of reaction rate, reduction in energy consumption and improvement of physical and mechanical properties of products [1,2,3]. Unfortunately, problems associated with the scaling-up of laboratory units to industrial capacities, such as hot spots and overheating effect, have limited the application of microwaves in the chemical industry [4,5]. To overcome these problems, it is essential to investigate the thermal analysis of microwave-heating chemical reactions, based on the multiphysics calculation.

Solving a fully time-transient coupled electromagnetic-thermal problem is very expensive, as the solution to the final set of equations is required at each time-step [6]. In the majority of industrial-heating problems, the electromagnetic dynamics are much faster than the thermal dynamics. On the time scale, the electromagnetic field is weakly coupled with a time-transient non-linear thermal field, and a weakly coupled electromagnetic-thermal model is adequate [7,8]. Therefore, in terms of the multiphysics calculation, it is possible to implement an iterative method based on a weak-coupling procedure. However, in a reaction process, certain components in the chemical reactions may be sensitive to the thermodynamic state, and chemical reactions may have a significant influence on the thermodynamic state [9]. Meanwhile, most chemical reactions are non-stationary media, which exhibit the time-varying characteristics because the component concentrations change with the reaction time [10]. Consequently, the degree of physical interaction between the electromagnetic field and the thermal field may be changed profoundly during the reaction processes. More importantly, the degree of coupling may affect the accuracy of the solution of an iterative method [11]. Unfortunately, little attention to detail and implications has been given to the multiphysics coupling between the electromagnetic field and the thermal field in microwave-heating chemical reactions.

In this paper, we investigate the degree of coupling in microwave-heating polar-molecule reactions. In the next section, the entropy-balance equation for microwave heating a polarized system is reviewed and the entropy-balance equation for microwave heating reactions is derived according to the energy-balance equation. Then, based on the entropy-source strength, the coupling degree of the electromagnetic field and thermal field can be given in terms of the phenomenological equations. Finally, we discuss the effects of the component-concentration variation on the coupling degree of the electromagnetic field and the thermal field in microwave-heating reactions.

## 2. Entropy-Balance Equation of Microwave Heating

### 2.1. In the Polarized System

For a non-reacting multicomponent-polarized-system with *n* components inside the microwave fields, the balance equation of internal energy can be described by the first law of thermodynamic, as [12,13]:

(1) $$\frac{du}{dt}=\frac{dq}{dt}-p\frac{dv}{dt}+E\cdot \frac{d(\rho^{-1}P)}{dt}+B\cdot \frac{d(\rho^{-1}M)}{dt}+\sum_{j=1}^{n}\mu_{j}\frac{dc_{j}}{dt},$$

where *u* is the specific internal energy, q is the heat added to a mass element of unit mass, p is the equilibrium pressure, v is the specific volume, and ρ is the mass density of the material; $\mu_{j}$ and $c_{j}$ are the chemical potential and the weight fraction of the *j*th component, respectively. ***E*** and ***B***, respectively, are the instantaneous electric and magnetic fields inside the system, which induce the local electric polarization, ***P***, and the local magnetic polarization, ***M***.

Although microwave heating is not at global equilibrium, it is assumed that there exists a state of local equilibrium in any small-mass element when the deviations from the equilibrium are not too large. For the hypothesis of local equilibrium, the local entropy is the same function as dq=Tds in equilibrium, in which s is the entropy per unit mass and *T* is the absolute temperature. $E_{eq}$ and $B_{eq}$ represent the instantaneous values of the electric and magnetic field at local equilibrium, respectively. Equation (1) can be expressed as

(2) $$\frac{ds}{dt}=\frac{1}{T}\frac{du}{dt}+\frac{p}{T}\frac{dv}{dt}-\frac{E_{eq}}{T}\cdot \frac{d}{dt}(\rho^{-1}P)-\frac{B_{eq}}{T}\cdot \frac{d}{dt}(\rho^{-1}M)-\sum_{j=1}^{n}\frac{\mu_{j}}{T}\frac{dc_{j}}{dt}.$$

Introducing Equation (2) into Equation (1), the entropy-balance equation in the polarized system can be expressed as

(3) $$\rho \frac{ds}{dt}=\frac{\rho }{T}\frac{dq}{dt}-\frac{1}{T}(E_{eq}-E)\cdot \frac{dP}{dt}--\frac{1}{T}(B_{eq}-B)\cdot \frac{dM}{dt}$$

### 2.2. In Polar-Molecule Reactions

We consider an isotropic mixture consisting of *n* components, amongst which *m* chemical reactions are possible. At constant pressure and temperature, the heat conduction and diffusion caused by uniform pressure or temperature may be neglected. Furthermore, we restrict ourselves to the diffusion process where the *j*th component concentration is non-uniform over the system. Under these conditions, based on the conservation of mass in chemical reactions and the diffusion process, the rate of the mass change of the *j*th component can be expressed as [14]

(4) $$\rho \frac{dc_{j}}{dt}=-\nabla \cdot J_{j}+\sum_{r=1}^{m}\nu_{jr}J_{r}.$$

$J_{j}$ is the diffusion flux of the *j*th component; $\nu_{jr}J_{r}$ is the production of the *j*th component per unit volume in the *r*th chemical reaction, where $J_{r}$ is the chemical-reaction rate of the *r*th reaction. In addition, $\nu_{jr}$ is proportional to the stoichiometric coefficient with which the *j*th component appears in the *r*th chemical reaction.

For chemical reactions and the diffusion process, with the addition of Equation (4), the rate of entropy change due to chemical reactions is then given as

(5) $$\rho \frac{ds}{dt}=-\rho \sum_{j=1}^{n}\frac{\mu_{j}}{T}\frac{dc_{j}}{dt}=\nabla \cdot \sum_{j=1}^{n}\frac{\mu_{j}}{T}J_{j}-\sum_{j=1}^{n}J_{j}\cdot \nabla \frac{\mu_{j}}{T}+\sum_{r=1}^{m}J_{r}\frac{a_{r}}{T},$$

where the chemical affinity of the *r*th reaction, $a_{r}$, is defined as $a_{r}=-\sum_{j=1}^{n}\mu_{j}\nu_{jr}(r=1,2,\cdots m)$ [15,16].

Finally, by merging Equations (5) and (3), in microwave-heating chemical reactions, the explicit form of the entropy-balance equation can be expressed as

(6) $$\rho \frac{ds}{dt}=\frac{\rho }{T}\frac{dq}{dt}-\frac{1}{T}(E_{eq}-E)\cdot \frac{dP}{dt}--\frac{1}{T}(B_{eq}-B)\cdot \frac{dM}{dt}+\nabla \cdot \sum_{j=1}^{n}\frac{\mu_{j}}{T}J_{j}-\sum_{j=1}^{n}J_{j}\cdot \nabla \frac{\mu_{j}}{T}+\sum_{r=1}^{m}J_{r}\frac{a_{r}}{T}.$$

## 3. The Heat Flow and Polarization Current of Microwave Heating in Polar-Molecule Reactions

The differential form of the energy-conservation principle defines the relationship between the heat added per unit of mass, q, and the heat flow, $J_{q}$, as

(7) $$\rho \frac{dq}{dt}=-\nabla \cdot J_{q}.$$

Inserting Equation (7) into Equation (6), in microwave-heating chemical reactions, we get

(8) $$\rho \frac{\partial s}{\partial t}=-\nabla \cdot (\frac{J_{q}-\sum_{j=1}^{n}\mu_{j}J_{j}}{T})-\frac{1}{T^{2}}J_{q}\cdot \nabla T-\frac{1}{T}\frac{\partial P}{\partial t}\cdot (E_{eq}-E)-\frac{1}{T}\frac{dM}{dt}\cdot (B_{eq}-B)-\sum_{j=1}^{n}J_{j}\cdot \nabla \frac{\mu_{j}}{T}+\sum_{r=1}^{m}J_{r}\frac{a_{r}}{T}.$$

As the magnetic permeability of chemical reactions is close to that of free space, the magnetic polarization ***M*** is not considered. Consequently, we obtain

(9) $$\rho \frac{\partial s}{\partial t}=-\nabla \cdot (\frac{J_{q}-\sum_{j=1}^{n}\mu_{j}J_{j}}{T})-\frac{1}{T^{2}}J_{q}\cdot \nabla T-\frac{1}{T}\frac{\partial P}{\partial t}\cdot (E_{eq}-E)-\sum_{j=1}^{n}J_{j}\cdot \nabla \frac{\mu_{j}}{T}+\sum_{r=1}^{m}J_{r}\frac{a_{r}}{T}.$$

According to the second law of thermodynamics [14],

(10) $$\rho \frac{\partial s}{\partial t}=-\nabla \cdot J_{s}+\Theta$$

where $J_{s}$ is the entropy flux per unit area and unit time, and Θ is the entropy-source strength. Comparing Equation (9) with Equation (10), the expressions for the entropy flux and the entropy production are given as

(11) $$J_{s}=\frac{1}{T}(J_{q}-\sum_{j=1}^{n}\mu_{j}J_{j})$$

and

(12) $$\Theta =J_{q}\cdot \frac{-\nabla T}{T^{2}}-\frac{1}{T}\frac{\partial P}{\partial t}\cdot (E_{eq}-E)-\sum_{j=1}^{n}J_{j}\cdot \nabla \frac{\mu_{j}}{T}+\sum_{r=1}^{m}J_{r}\frac{a_{r}}{T}.$$

Furthermore, if chemical reactions have uniform density and the diffusion flux $J_{j}$ is neglected, Equation (12) reduces to

(13) $$\Theta =J_{q}\cdot \frac{-\nabla T}{T^{2}}-\frac{1}{T}\frac{\partial P}{\partial t}\cdot (E_{eq}-E)+\sum_{r=1}^{m}J_{r}\frac{a_{r}}{T}.$$

To further investigate the influence of the reaction process on the heat and polarization without loss of generality, we assume that chemical reactions are homogeneous isotropic media without free charges and currents, and only the *j*th component has a polarization $P=\phi_{j}*[c_{j}E]$. $\phi_{j}$ and $c_{j}$ are the rotational-diffusion function and concentration of the *j*th component, respectively; ∗ represents the convolution; $\phi_{j}=\varepsilon_{0}\chi \exp(-t/\tau )/\tau$, where χ is the electric susceptibility, $\varepsilon_{0}$ is the vacuum permittivity and τ is the dielectric relaxation time [10]. Accordingly, the differential form of polarization is $\partial P/\partial t=-(P-\varepsilon_{0}\chi c_{j}E)/\tau$, where ∂P/∂t represents the polarization current, $J_{p}$, and we can get $E_{eq}=P/(\varepsilon_{0}\chi c_{j})$. Equation (13) becomes

(14) $$\Theta =J_{q}\cdot \frac{-\nabla T}{T^{2}}-\frac{1}{T}J_{p}\cdot \frac{\left(P-\varepsilon_{0}\chi c_{j}E\right)}{\varepsilon_{0}\chi c_{j}}+\sum_{r=1}^{m}J_{r}\frac{a_{r}}{T}.$$

When uniform temperature is attained (∇T=0) and the reactions are at equilibrium (the chemical affinity, $a_{r}$, vanishes), we have

(15) $$\Theta =-\frac{1}{T}\frac{\partial P}{\partial t}\cdot \frac{\left(P-\varepsilon_{0}\chi c_{j}E\right)}{\varepsilon_{0}\chi c_{j}}=\frac{1}{T}\frac{\tau }{\varepsilon_{0}\chi c_{j}}{\left|\frac{\partial P}{\partial t}\right|}^{2}.$$

Specifically, the term $\frac{\tau }{\varepsilon_{0}\chi c_{j}}{\left|\frac{\partial P}{\partial t}\right|}^{2}$ on the right-hand side denotes the power converted into local entropy production by electro-thermal coupling, which corresponds to the transient power-loss-density of electromagnetic waves in the reactions reported by Liao et al. [17].

Based on the phenomenological equations and Curie–Prigogine principle, Equation (14) leads to the related phenomenological equations

(16) $$J_{q}=-L_{qq}\frac{\nabla T}{T^{2}}-L_{qp}\frac{\left(P-\varepsilon_{0}\chi c_{j}E\right)}{T\varepsilon_{0}\chi c_{j}},$$

(17) $$J_{p}=-L_{pq}\frac{\nabla T}{T^{2}}-L_{pp}\frac{\left(P-\varepsilon_{0}\chi c_{j}E\right)}{T\varepsilon_{0}\chi c_{j}}$$

and

(18) $$J_{r}=\sum_{h=1}^{m}l_{rh}\frac{a_{h}}{T}\begin{array}{cc} & \left(r=1,2,\cdots m\right) \end{array}.$$

Here, the phenomenological coefficients $L_{qq}$, $L_{qp}$, $L_{pq}$, $L_{pp}$ and $l_{rh}$, which are scalar quantities in isotropic media, represent the interactions between the various processes. Equations (16) and (17) describe the vectorial cross-effects of the heat flow and polarization current, and Equation (18) denotes the scalar processes of the *r*th chemical reactions and their possible cross-phenomena. Referring to the Curie–Prigogine principle, a scalar process cannot produce a vectorial change. More generally, chemical affinity cannot cause the directed heat flux and polarization current in the isotropic reactions.

After comparing Equations (16) and (17) with Fourier’s law $J_{q}=-k\nabla T$ and the differential form of the polarization expression, where *k* is the thermal conductivity, we get

(19) $$L_{qq}=kT^{2}$$

and

(20) $$L_{pp}=T\varepsilon_{o}\chi c_{j}/\tau .$$

Then, the coefficients $L_{pq}$ and $L_{qp}$ are expressed by [18]

(21) $$L_{pq}=\frac{\partial J_{p}}{\partial (\nabla (1/T))}$$

and

(22) $$L_{qp}=\frac{\partial J_{q}}{\partial \left(\tau /(T\varepsilon_{o}\chi c_{j})J_{p}\right)}.$$

The cross-phenomenological coefficient represents the coupling of the heat flow and polarization current in isotopic media; namely, the polarization current arises from the thermal diffusion. According to the Onsager reciprocal relation, the coefficient $L_{qp}$ is equal to $L_{pq}$, that is $L_{qp}=L_{pq}$.

The final expressions for the heat flow and polarization current are then expressed as

(23) $$J_{q}=-k\nabla T-L_{qp}\frac{\left(P-\varepsilon_{0}\chi c_{j}E\right)}{T\varepsilon_{0}\chi c_{j}}$$

and

(24) $$J_{p}=-L_{pq}\frac{\nabla T}{T^{2}}-\frac{\left(P-\varepsilon_{0}\chi c_{j}E\right)}{\tau }.$$

Equation (23) indicates that the heat flow is determined by both thermal conduction and polarization, and quantitatively describes a particular excess effect due to polarization loss. In fact, in the absence of a thermal gradient, that is ∇T=0, the heat flow arises from the power loss of the electromagnetic waves. On the other hand, Equation (24) suggests that the polarization current is influenced not only by the instantaneous polarization and electric field, but also by the thermal gradient. Here, we suppose that the instantaneous electric field, ***E***, is zero, and therefore P=0. Thus, Equation (24) implies that the polarization current can flow in non-uniformly heated reactions even if the electric field, ***E***, is zero. This can be explained by the directional motion of the polar molecules due to the thermal gradient, namely the thermal diffusion.

## 4. The Coupling Degree of the Electromagnetic Field and Thermal Field

Kedem and Caplan have defined the degree of coupling, $\lambda_{qp}$, as

(25) $$\lambda_{qp}=\frac{L_{qp}}{{\left(L_{qq}L_{pp}\right)}^{1/2}}$$

which has been used as a basis for comparison of systems with various coupled flows [19,20]. By using Equations (19), (20) and (22), the degree of coupling can be expressed as

(26) $$\lambda_{qp}=\frac{\partial J_{q}}{\partial \left(\tau /(T\varepsilon_{o}\chi c_{j})J_{p}\right)}\frac{\sqrt{\tau }}{T\sqrt{kT\varepsilon_{o}\chi c_{j}}}.$$

Equation (26) shows that $\lambda_{qp}$ is directly proportional to the rate of $J_{q}$ variation, and inversely proportional to the rate of $J_{p}$ variation.

The reactions are proofed to exhibit the time-varying and dispersive characteristics. The time-varying characteristics originate from the changes in component concentration. When the time scale of the component-concentration variation is greater than the wave period, adiabatic approximations can be used to neglect transients [10]. The reactions can be considered as general dispersive media, in which on the time scale, the rate of $J_{q}$ variation is much smaller than the rate of change in $J_{p}$. For this situation, based on Equation (26), $\lambda_{qp}$ is usually sufficiently small. Therefore, the influence of the reaction processes on the degree of coupling can be ignored. Furthermore, the heat flow and polarization current are independent of each other, especially when $\lambda_{qp}$ is approximately close to zero. Thus, in the majority of microwave-heating problems, electric and thermal fields are considered as weakly coupled.

On the other hand, while the time scale of the component-concentration variation is smaller than the wave period, that is, the component concentration changes rapidly with time, transients become apparent, and frequency changes in the electromagnetic field can be observed [10]. The effect of time-varying characteristics on the polarization current $J_{p}$ is much more obvious, and the rate of change in $J_{p}$ changes rapidly with time. This means that the degree of coupling may vary during the reaction processes. Thus, the effects of the reaction processes on the degree of coupling are noticeable. Most importantly, if the rate of change of $J_{p}$ decreases in the reactions, the reduction leads to the increase in $\lambda_{qp}$. The higher the absolute value of $\lambda_{qp}$, the higher the dependence of the heat flow on the polarization current, thereby provoking a transition from weak coupling to strong coupling. More importantly, the degree of coupling may affect the accuracy of the numerical solution as well as the computational efficiency, which should be noticed carefully in the simulation of microwave-assisted chemical reactions.

All in all, the coupled conditions under different reaction processes exhibit various degrees of interaction between the electromagnetic and thermal fields. Generally, in the microwave-heating process, the time scale of the component-concentration variation is greater than the wave period. Hence, the electromagnetic-thermal models are considered as weakly coupling, and it is possible to implement an iterative method based on a weak-coupling procedure [21,22]. On the other hand, when the time scale of the component-concentration variation is smaller than the wave period, the interaction changes profoundly. Classical examples are frequency conversions in the rapidly created plasma [23,24]. Thus, in terms of the multiphysics calculation, numerical calculations need to be converted into strong-coupling algorithms.

## 5. Conclusions

In this paper, we have derived the coupling degree of the electromagnetic field and the thermal field in microwave-heating polar-molecule reactions, based on the entropy-balance equation. Because of the time-varying characteristics of the reactions, the effects of the reaction processes on the degree of coupling are discussed. If the time scale of the component-concentration variation in reaction processes is much greater than the wave period, the degree of coupling approaches zero and there is a weak coupling between the electric and thermal fields. On the other hand, when the time scale of the component-concentration variation is smaller than the wave period, the degree of coupling may vary during the reaction processes. When the absolute value of the degree of coupling becomes larger, there may be a change from weak coupling to strong coupling.

## Data Availability

Data will be made available on request.
